# Development of a Microwave Sensor for Real-Time Monitoring of a Micro Direct Methanol Fuel Cell

**DOI:** 10.3390/s24030969

**Published:** 2024-02-02

**Authors:** Shubin Zhang, Tian Qiang, Yanfeng Jiang

**Affiliations:** 1 School of Internet of Things Engineering, Jiangnan University, Wuxi 214122, China; shubinzhang@jiangnan.edu.cn (S.Z.); qtknight@jiangnan.edu.cn (T.Q.); 2 Institute of Advanced Technology, Jiangnan University, Wuxi 214122, China

**Keywords:** microwave sensor, nonintrusive detection, online monitoring, micro direct methanol fuel cell (μDMFC), methanol crossover

## Abstract

Micro direct methanol fuel cells (μDMFCs) are a promising power source for microelectronic devices and systems. As the operating state and performance of a μDMFC is generally determined by both electrochemical polarization and methanol crossover, it is important to monitor the methanol concentration in μDMFCs. Here, we design and fabricate a microwave sensor and integrate it with a μDMFC for the online detection of methanol concentration in a nonintrusive way. The sensing area is set at the bottom of the anode chamber of a μDMFC which exhibits a maximum output power density of 28.8 mW cm^−2^ at 30 °C. With a square ring structure, the dual-mode microwave sensor shows a sensitivity of 9.5 MHz mol^−1^ L. Furthermore, the importance of methanol concentration monitoring is demonstrated in the long term. A relatively smooth methanol decline curve was obtained, which indicated a normal and stable operating status of the μDMFC. Derived from real-time recording data, fuel utilization was additionally calculated as 28.5%.

## 1. Introduction

In the past decade, micro fuel cells have received lots of attention in the electronic engineering field due to not only the world’s growing energy crisis but also the urgent need for micro power sources for highly integrated microelectronic devices and systems [1,2,3]. Among novel technologies, micro direct methanol fuel cells (μDMFCs) have shown great advantages of light weight, safety and reliability, environmental friendliness, high energy density, and ease of carrying and use [4,5]. However, two major factors still hinder the prospect of the commercialization of μDMFC technology. One is the low reaction kinetics of methanol and water in room temperature and the other is that the commonly used membrane, namely Nafion membrane, is not able to prevent methanol fuel from crossover. For both factors, extensive research shows that methanol concentration plays such an important role that increasing fuel concentration promotes the methanol oxidation reaction rate but inevitably increases methanol crossover. This is due to the relatively high methanol permeability of the Nafion membrane [6]. Up till now, a clear majority of μDMFCs have still operated with low concentrations of methanol; therefore, raising methanol concentration in μDMFCs is of great interest but also faces great challenges [7,8,9,10]. In addition, μDMFCs have to be stacked to obtain a sufficiently high output voltage for electronic devices or systems. Multiple-cell packaging for micro fuel cells is highly difficult and there is a huge potential risk of fuel leakage. For those μDMFCs aiming to be supplied with high-concentration methanol, an unexpected leak or heavy crossover of methanol can cause more damage. Therefore, given the fact that the performance of a μDMFCs is so sensitive to methanol concentration, it is necessary to detect it in system monitoring, and therefore the online monitoring of methanol concentration should be a must-have function in future μDMFC devices.

Traditional chemical and physical methods are commonly used for solute concentration detection. Although the chemical titration method is easy to conduct for ion concentration detection, it is not suitable for methanol concentration detection in fuel cells. Alternatively, one of the typical physical methods is gas chromatography. It was used to measure methanol concentration in a study of preventing methanol crossover in DMFCs [11]. Unfortunately, this method contains a step of obtaining aqueous solutions from the fuel cell, either by a micropipette or a syringe, which makes it an intrusive way. However, in well-assembled fuel cell stacks, it is almost impossible to obtain a solution sample when in operation. Therefore, the online detection of fuel concentration in DMFCs needs noncontact and nonintrusive methods. In recent years, nonintrusive and online ways of solute concentration measurements have been in great demand, not only in the fuel cell industry, but also in the medical and pharmaceutical industry. In addition, real-time monitoring of fuel cell operating status should also be mentioned and carried out. Methanol concentration, as the main index of the performance of a DMFC, needs to be monitored constantly, besides other parameters like temperature, output voltage, etc.

Nowadays, microwave sensors based on radio frequency (RF) resonance have shown the ability to accurately quantify aqueous solutions in noncontact ways and have received great attention in areas like chemistry, food, and biological medicine. These sensors can be introduced into fuel cell systems for fuel concentration monitoring [12,13,14]. In common cases, it operates with advantages like multidimensional detection-based enhanced accuracy, real-time operation, reusability, contactless corrosive materials, and lab-on-a-chip compatibility. To take advantage of these attractive features, the sensing region for the interaction between the material under test and the electromagnetic wave should be carefully designed. Since the resonant mode design is critical to the sensitivity and stability of the microwave sensor, it is commonly seen that the sensitivity of an RF resonance sensor is approximated using the product of loaded quality and filling factors.

In this paper, we exploit a nonintrusive, online way of methanol concentration detection and operation status monitoring for μDMFCs. The proposed RF sensor is based on a square ring resonator. The integration of the sensor with the μDMFC guarantees a stable sensitivity of the RF resonance sensor and makes the device more compact. Besides the primary function of the sensor, i.e., the online detection of fuel concentration, it also makes it easy to access the real-time record of the operation status of the μDMFC in case of fuel leakage or abnormal fuel crossover. Fuel utilization, often regarded as the performance index for fuel cells, can also be calculated for any long-term discharging process.

## 2. Materials and Methods

**Figure 1:**
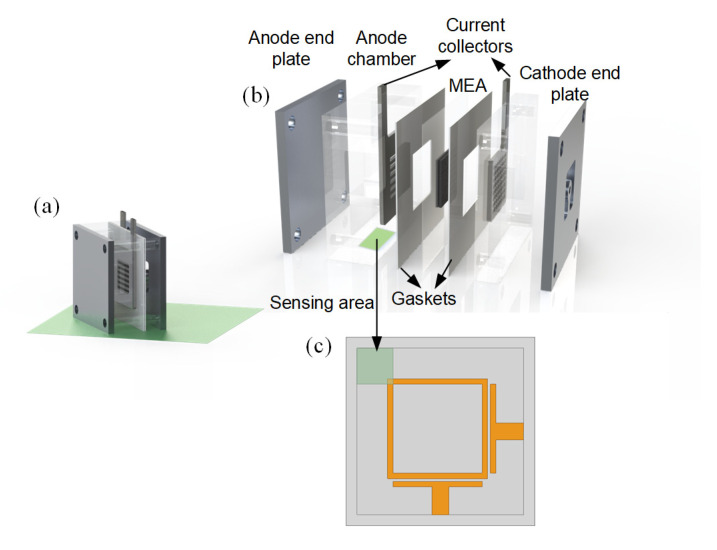
(**a**) Schematic of a sensor-integrated μDMFC, (**b**) key components of the μDMFC, and (**c**) layout of the RF resonance sensor.

Figure 1 shows the schematic of the proposed sensor-integrated μDMFC, the key components of the μDMFC, and the layout of the RF resonance sensor. As can be seen from the figures, the multilayer structure of the μDMFC consists of a cathode end plate, a cathode current collector, a membrane electrolyte assembly (MEA), an anode current collector, and an anode chamber. The MEA, with multiple porous layers, catalytic structures, and a proton exchange membrane in between, has an active area of 1 cm^2^. The RF resonance sensor is attached to the bottom of the μDMFC; thus, the sensing area is directly below the anode chamber. Originally, the anode chamber had a volume of about 1 cm^3^ for fuel, namely methanol aqueous solution. But in order to keep the fuel close to the sensor, we intentionally hollowed out a 6 × 6 mm^2^ area at the bottom of the anode chamber so the fuel could be located closer to the sensor.

The RF sensor, based on a square ring resonator in which the two ports are orthogonal, is shown in Figure 1c. The length difference between the two transmission paths from port 1 to port 2 equals π. Restrained by the placement of the ports, the resonator is not able to split the degenerate modes; therefore, a small chamber containing the methanol solution is placed in the 45° azimuth, which is defined as the sensing region. By forming a perturbation in the ring resonator, the balance of the resonator is destroyed. As a result, the coupling between the degenerate modes in the cavity of the square ring resonator becomes the key to generating dual-mode resonance. In order to implement the proposed RF resonance sensor with miniaturized size and enhanced filling factor, the side length and the line width of the square ring are set to 16 mm and 1 mm, and the line length and width of the interfacial part are 6.5 mm and 4 mm. It should be noted that the size of the resonator matches the size of a μDMFC, which is useful for further easy integration.

The sensor utilizes the parallel relationship of methanol concentration and dielectric properties of the methanol solution. When the concentration of the methanol solution changes, the dielectric constant changes accordingly. As a result, the resonant frequency of the RF sensor, which is based on near-field electromagnetic wave resonance, is affected by the change of the dielectric properties. With dual-mode resonance, the generated lower central frequency (*f*_L_) of the square ring resonator will change accordingly with methanol concentration and can conveniently be measured as S-parameters. The penetration depth of the concentrated electromagnetic wave in the sensing region oscillating at the resonator’s *f*_L_, which is a measure of how deeply RF radiation can penetrate the methanol chamber and sense methanol concentration-based permittivity variations, also depends on both complex permittivity and temperature. It is calculated according to the following equation:

(1) $$D_{p}=\frac{\lambda }{2\pi {\left(2\varepsilon^{\prime }\right)}^{1/2}}{\left\{\left[1+{\left(\frac{\varepsilon^{\prime \prime }}{\varepsilon^{\prime }}\right)}^{2}\right]-1\right\}}^{-1/2}$$

where λ refers to the wavelength of the RF electromagnetic wave in free space.

**Figure 2:**
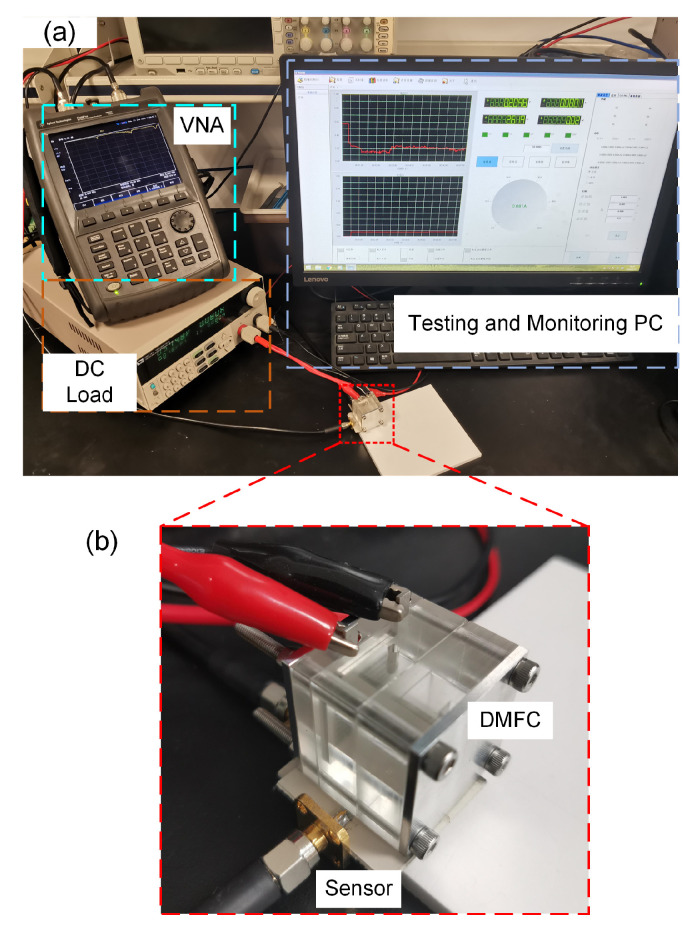
Schematic of (**a**) the experimental setup and (**b**) the proposed device of a self-breathing transparent μDMFC integrated with an RF resonance sensor.

The proposed device of a self-breathing transparent μDMFC integrated with an RF resonance sensor was fabricated in order to verify the simulation results. First and foremost, a piece of 5-layered MEA with 1 cm^2^ active area was fabricated using the catalyst-coated-membrane (CCM) method. We employed a Pt-Ru/C catalyst of 4.0 mg cm^−2^ loading on the anode and Pt/C catalyst of 2.0 mg cm^−2^ on the cathode, with a Nafion117 membrane in between. Prior to the experimental tests, the following activation process was conducted at a temperature of 60 °C: Firstly, deionized water was fed into the anode chamber of the μDMFC to humidify the MEA for over 2 h. Secondly, the fuel cell was maintained at a discharging current density of 3 mA cm^−2^ for 2 h with 2.0 M methanol solution. Thirdly, the fuel cell operated with a high current density of 30 mA cm^−2^ until the output voltage reached its maximum value. The current collectors, made of 316 stainless steel, were fabricated by a computer numerical control (CNC) machine. The anode current collector was designed with a grid shape while the cathode current collector had a perforated structure. The anode chamber, made of organic glass, has a volume of 2 × 1.2 × 1.2 cm for methanol solution. In addition, silicone gaskets were fabricated by laser cutting to provide good sealing between the anode chamber, current collectors, and the MEA. Al-based endplates furthermore guaranteed good sealing and electrical contact of the fuel cell’s essential components. Figure 2 illustrates the experimental setup for testing and monitoring. To conduct the electrical tests and measurements, the RF resonance sensor and the μDMFC were connected to an Agilent N9923A RF vector network analyzer (VNA) (Agilent Technologies, Inc., Santa Clara, CA, USA) and an Itech IT8511A+ DC electronic load (Itech Electronic co., Ltd., Nanjing, China), respectively. To automatically monitor the μDMFC in real time, both the VNA and the programmable DC load were connected to a personal computer (PC) through a network and RS232 protocol, respectively. Every 10 s, the status of the μDMFC was recorded and saved in the PC. An enlarged picture of the assembled μDMFC on top of the RF sensor is shown in Figure 2b. The proposed RF resonance sensor with optimized dimensions was fabricated on a 0.508 mm thick substrate with a dielectric constant of 2.52 and a loss tangent of 0.002 using the wet etching printed circuit board (PCB) technique. The side length of the squared substrate is 32 mm. Both the top and bottom of the substrate was covered with a 0.018 mm thick copper layer with a conductivity of 5.8 × 10^7^ S·m. The fabricated sensor was embedded under the anode chamber of the μDMFC and was fixed to the aluminum end plate. 

## 3. Results and Discussion

### 3.1. Numerical

**Figure 3:**
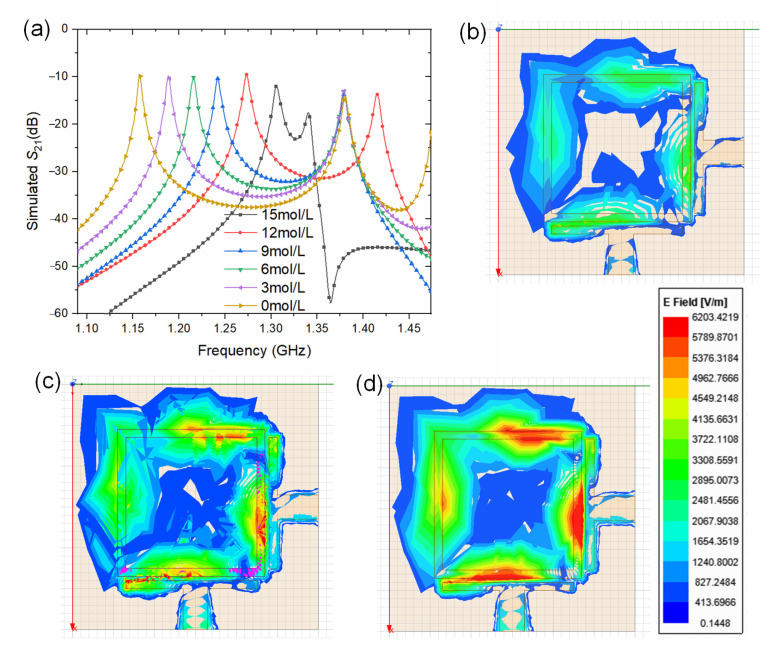
Simulation results of (**a**) the central frequencies indicated by peaks of S21 of the RF sensor, (**b**) E-field distribution at 1.23 GHz without load, (**c**) E-field distribution at 1.04 GHz without load, and (**d**) E-field distribution at 1.23 GHz with 6 mol L^−1^ methanol solution.

As indicated by the layout of the RF sensor, dual mode refers to the fact that the square ring resonator is equivalent to a parallel connection of two half-wavelength resonators. The resonant frequency of the square ring resonator with either one port or two ports shall be the same. The computing software HFSS was employed in the simulation process. The resulting central frequencies are indicated by the peaks of *S*_21_. Therefore, the S-parameters of the proposed RF sensor were calculated with methanol solution samples of various concentrations (0 to 15 mol L^−1^). Temperature was set at 30 °C, and the simulation results are shown in Figure 3a. It is seen from the figure that the central frequencies of the dual-mode resonator sensor vary along with the methanol concentration. It can be explained that the increase in the methanol solution induces the dielectric constant to decline, and then affects not only the frequency of the degenerate mode, but also the coupling coefficient between two split modes. When the methanol concentration increases from 0 to 12 mol L^−1^, the lower values of the peaks exhibit a near-linear relationship between concentration and resonance frequency. From the simulation results, it can be inferred that the sensitivity of the RF sensor is about 9.9 MHz mol^−1^ L. It should also be noted that with a higher methanol concentration (over 12 mol L^−1^), the relationship between *f*_L_ and methanol concentration exhibits very low linearity. Nevertheless, the test scope of the sensor already covers the operating fuel concentration for common μDMFCs. E-field distributions above the sensing region with and without load are also shown in Figure 3. First, the simulation condition is set as the bare sensor without load at 1.23 GHz. The result shown in Figure 3b reveals a nonresonant state, at 1.23 GHz, leading to a significant energy loss. The resonance frequency of the sensor without load is 1.04 GHz, and the resonant state is shown in Figure 3c. In Figure 3d, the sensor is loaded with 6 mol/L methanol solution and the excitation is set at a resonance frequency of 1.23 GHz. The figure shows the E-field of the resonant state, in which the maximum E-field is about 6200 V/m. 

### 3.2. Experimental

**Figure 4:**
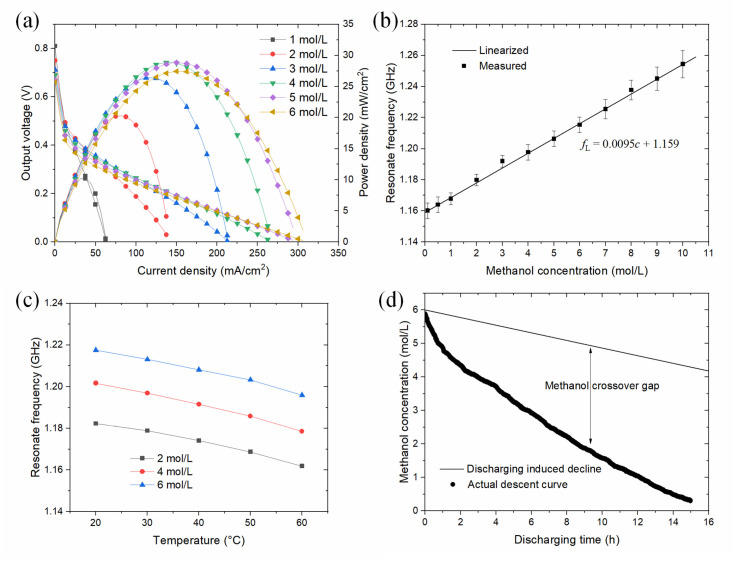
(**a**) Performance of the μDMFC with various methanol concentrations, (**b**) resonance frequency as a function of methanol concentration, (**c**) effect of temperature on resonance frequency, and (**d**) real-time record of methanol concentration during a long run.

The performance of the μDMFC with different methanol concentrations is depicted in Figure 4a. The discharge tests were conducted at a constant temperature of 30 °C. The results indicate that the best performance, of about 28.0 mW cm^−2^, was achieved when the fuel cell was supplied with a methanol concentration of 4 to 5 mol L^−1^. For lower methanol concentrations, it is easy to understand that the performance of the fuel cell dropped dramatically due to insufficient reaction rate. But for a higher methanol concentration, 6 mol L^−1^ in this case, the maximum output power decreased to about 25.9 mW cm^−2^. This can be explained by the fact that the unwanted methanol crossover mechanism degraded the fuel cell’s performance by causing a huge potential loss for the oxygen reduction reaction in the cathode. Methanol crossover flux is proportional to methanol concentration in the anode side of the MEA; thus, a tradeoff has to be made between improving the reaction rate and decreasing fuel crossover. This explains why the best performance for this μDMFC appeared when the methanol concentration was no more than 5 mol L^−1^. Nevertheless, a 6 mol L^−1^ methanol concentration can still be used as we move to the following test of a long-term discharging process.

The developed sensor was applied to accurately quantify a wide range of methanol concentrations in aqueous solutions. Firstly, a series of tests were conducted for methanol solutions with the concentration range from 0 to 10 mol L^−1^. The solutions were kept at room temperature (24.5~25.3 °C) after preparation. *S*-parameters were measured and central frequencies were determined for the resonator. As was analyzed in the numerical section, the resulting central frequencies were indicated by the peaks of *S*_21_. Shown in Figure 4b, the measured *f*_L_ (GHz) is correlated with methanol concentration (*c*, mol L^−1^) as:

(2) $$f_{L}=0.0095c+1.159$$

Therefore, according to the linearized curve, the RF resonance sensor provided a sensitivity of about 9.5 MHz mol^−1^ L for the methanol aqueous solution. After the optimal methanol concentration for the fuel cell and the sensitivity of the RF sensor were determined, the effects of temperature on resonance frequency were studied and are summarized in Figure 4c. Concentrations ranging from 2 to 6 mol L^−1^ were tested under a temperature variation from 20 to 60 °C. In general, a unidirectional negative correlation between *f*_L_ and sample temperature was found. The temperature-dependent shift exhibited an average sensitivity of −0.638 MHz °C^−1^. In fact, it can be approximately classified as a linear change. With respect to the temperature variation of the methanol concentration, Equation (2) should be modified to the following calibration equation:

(3) $$f_{L}=0.0095c+0.000638(30-T)+1.159$$

Based on the above investigations, it can be concluded that methanol concentration up to 6 mol L^−1^ was suitable for not only the operation of this μDMFC but also for the detection range of the RF sensor with a high and stable sensitivity. Furthermore, the results of the stability test, which can also be regarded as a long-term demonstration of noncontact sensing and online monitoring for the integrated μDMFC device, are shown in Figure 4d. The test was also a verification for the above numerical study. Prior to the discharging process, the anode chamber of the micro fuel cell was filled with 4.2 mL (almost the volume of the original anode chamber plus the sensing region) methanol solution of 6 mol L^−1^. Through DC load regulation, the discharging current was set to 5 mA for over 15 h. The actual methanol decline curve and the discharging-induced decline curve of methanol concentration are both shown. The actual methanol decline curve is relatively smooth, which indicates a normal and stable operation of the μDMFC. The recorded data also showed that there existed an enormous gap between the discharging loss and the actual loss of methanol, indicating that fuel loss induced by methanol crossover was more than four times over normal consumption. Using real-time methanol concentration values, the fuel utilization of this μDMFC was calculated after a long run. However, to our disappointment, it was only 28.5% in the end. Nevertheless, the functionality of this sensor-integrated μDMFC device was demonstrated through accurate nonintrusive detection and stable online monitoring.

The performance of the proposed RF sensor is compared with the ones reported in the literature, as listed in Table 1. As the status of fuel or the state of charge need to be monitored or calculated in engines, batteries, and fuel cells, our work uses an RF sensor to monitor fuel status in DMFCs for the first time. As can been seen from Table 1, this RF sensor-based method is more accurate than calculating fuel concentration based on the generated electric energy, since methanol crossover in DMFCs can hardly be accurately predicted. Without liquid transfer or any other invasive operation, it is also more cost-effective and convenient than traditional gas chromatography. Moreover, this RF sensor, with a conformal design, shows its potential of good and easy integration with micro fuel cells. In the future, an interfacial circuit for the RF sensor is needed, which can further simplify the whole monitoring system.

## 4. Conclusions

In summary, in this work, a newly proposed technique of noncontact sensing and nonintrusive monitoring was demonstrated to be highly efficient in novel fuel cells by integrating a liquid-fed μDMFC with a high-sensitivity RF resonance sensor. Firstly, based on a dual-mode square ring resonator, the RF sensor was designed and numerically verified to be capable of accurately quantifying physiologically relevant concentrations of methanol in the anode chamber of a μDMFC. Secondly, a μDMFC with an active area of 1 × 1 cm^2^ and a maximum power density of 28.8 mW cm^−2^ at 30 °C was fabricated as well as the proposed RF sensor. Their integration was achieved by hollowing out the bottom of the anode chamber, which then acted as the sensing area for the sensor. The experimental results revealed a sensitivity of 9.5 MHz mol^−1^ L for the RF sensor, which was slightly smaller than the simulated data. Moreover, a linear and unidirectional negative correlation between *f*_L_ and sample temperature enabled the proposed sensor to accurately quantify methanol concentration under a wide range of temperature variations. Finally, a long-term discharging operation was conducted through a demonstration of noncontact sensing and online monitoring for the integrated μDMFC device. Real-time fuel utilization was recorded, but to our disappointment, it was only about 28.5% in the end. The recorded data showed that there existed an enormous gap between the discharging loss and the actual loss of methanol, which was induced by methanol crossover. In the future, not only higher sensitivity of the sensor should be achieved, but also the compactness of the sensor-integrated fuel cell system should be improved.

## Figures and Tables

**Table 1:** Performance comparison with other concentration-measuring methods in the literature.

Ref.	Method	Solvent	Sensitivity	Features
This work	RF sensor	Methanol	9.5 MHz mol^−1^ L	Noninvasive
[13]	RF sensor	Uric acid	2.762 MHz mg^−1^ dL	Noninvasive
[15]	Gas chromatography	Methanol	±5%	Invasive, pipette
[16]	Calculation	Methanol	±30%	Noninvasive, no detector

## Data Availability

Data are contained within the article.
